# A revised nomenclature for the lemur family of protein kinases

**DOI:** 10.1038/s42003-023-05671-8

**Published:** 2024-01-08

**Authors:** Gábor M. Mórotz, Neil A. Bradbury, Oana Caluseriu, Shin-ichi Hisanaga, Christopher C. J. Miller, Agnieszka Swiatecka-Urban, Heinz-Josef Lenz, Stephen J. Moss, Georgios Giamas

**Affiliations:** 1 Department of Pharmacology and Pharmacotherapy, Semmelweis University, 1089 Budapest, Hungary; 2 Department of Physiology and Biophysics, Chicago Medical School, North Chicago, IL 60064 USA; 3 Department of Medical Genetics, University of Alberta Hospital, Edmonton, AB T6G 2H7 Canada; 4 Laboratory of Molecular Neuroscience, Department of Biological Sciences, Graduate School of Science, Tokyo Metropolitan University, Minami-Osawa, Hachioji, Tokyo, 92-0397 Japan; 5 Department of Basic and Clinical Neuroscience, Institute of Psychiatry, Psychology and Neuroscience, King’s College London, London, SE5 9RX UK; 6 Department of Pediatrics, University of Virginia School of Medicine, Charlottesville, VA 22903 USA; 7 Department of Medicine, University of Southern California/Norris Comprehensive Cancer Centre, Los Angeles, CA 90033 USA; 8 Department of Neuroscience, Tufts University School of Medicine, Boston, MA 02111 USA; 9 Department of Neuroscience, Physiology and Pharmacology, University College London, London, WC1 6BT UK; 10 Department of Biochemistry and Biomedicine, School of Life Sciences, University of Sussex, Brighton, BN1 9QG UK

**Keywords:** Cancer genetics, Tumour biomarkers, Alzheimer's disease, Protein transport, Phosphorylation

## Abstract

The lemur family of protein kinases has gained much interest in recent years as they are involved in a variety of cellular processes including regulation of axonal transport and endosomal trafficking, modulation of synaptic functions, memory and learning, and they are centrally placed in several intracellular signalling pathways. Numerous studies have also implicated role of the lemur kinases in the development and progression of a wide range of cancers, cystic fibrosis, and neurodegenerative diseases. However, parallel discoveries and inaccurate prediction of their kinase activity have resulted in a confusing and misleading nomenclature of these proteins. Herein, a group of international scientists with expertise in lemur family of protein kinases set forth a novel nomenclature to rectify this problem and ultimately help the scientific community by providing consistent information about these molecules.

## Abstract (web summary)

Here a group of scientists with expertise in lemur family of protein kinases propose a new nomenclature for lemur kinases.

## Introduction

Lemur kinase protein family consists of four protein members, lemur tyrosine kinase (LMTK) 1A, LMTK1B, LMTK2 and LMTK3, encoded by three genes. Despite their misleading name, work to date (published and unpublished) has predominantly demonstrated that they are actually serine/threonine-protein kinases instead of tyrosine kinases.

**Fig. 1:**
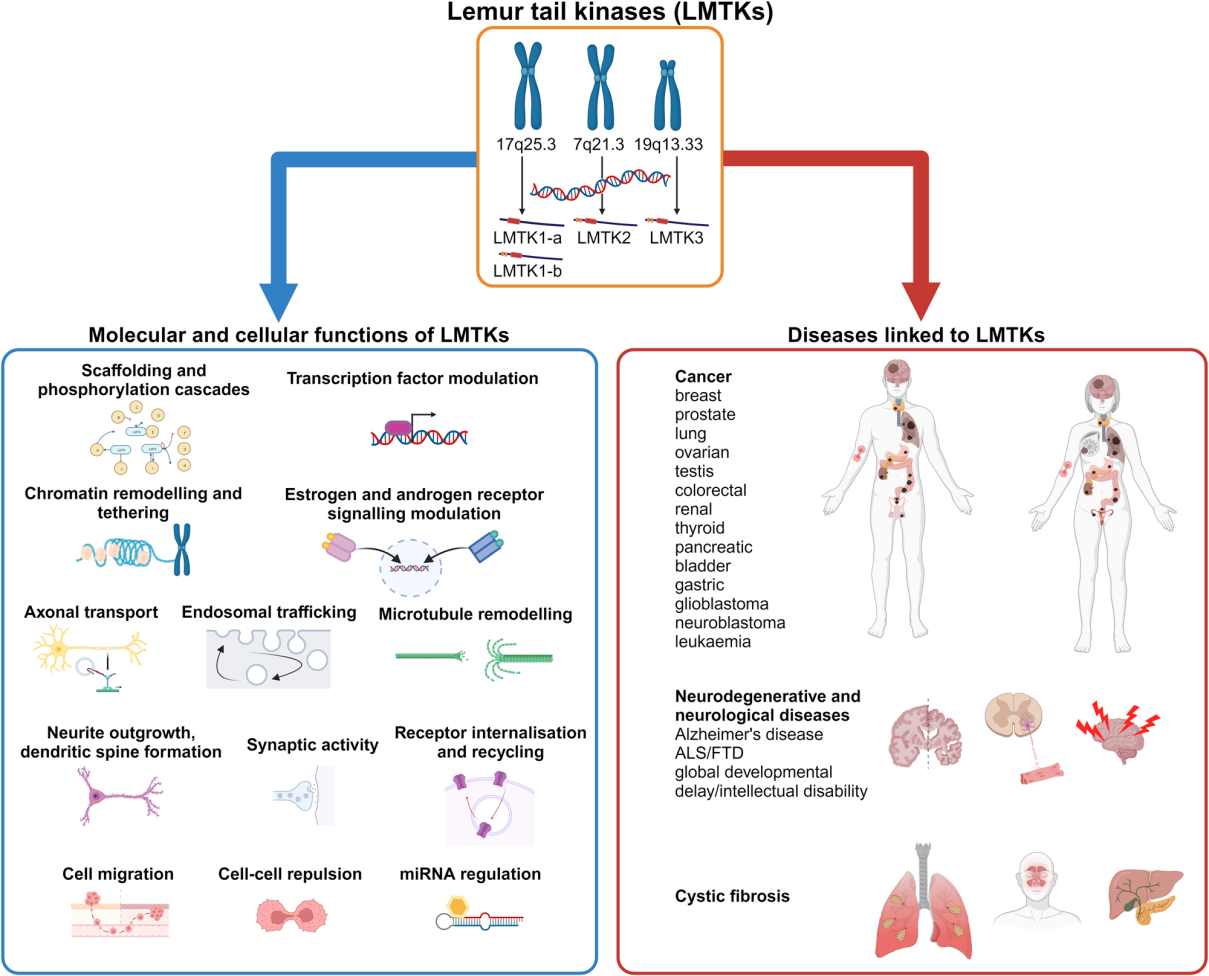
Molecular and cellular functions, and diseases associated with lemur tail kinases (LMTKs). LMTK1-a, LMTK1-b, LMTK2 and LMTK3 are involved in a wide range of cellular processes including gene expression regulation, protein phosphorylation cascades, intracellular transport, neuronal outgrowth, synaptic activity, and cell motility. LMTKs are also strongly implicated in the development and progression of a wide range of cancers, neurodegenerative and neurological diseases, and cystic fibrosis. ALS/FTD, amyotrophic lateral sclerosis/frontotemporal dementia; miRNA, microRNA.

During the last decade, LMTKs have gained increasing attention due to their involvement in various diseases including cancer, cystic fibrosis, Alzheimer’s disease, amyotrophic lateral sclerosis/frontotemporal dementia and global developmental delay/intellectual disability^1–6^ (Fig. 1). Their association with cancer is the most studied to date as they have been implicated in a wide range of tumours including breast, prostate, lung, colorectal, renal, testis and ovarian, thyroid, pancreatic, bladder, gastric, glio- and neuroblastoma, and leukaemia^7–9^.

Among them, *LMTK2* has been identified as a susceptibility gene in prostate cancer and several other studies also highlighted that changes in LMTK2 and LMTK3 expressions can also be used as prognostic markers in colorectal, gastric, prostate and breast cancers^4,5,10–12^. These oncogenic changes in LMTK expression can also promote tumour growth by modulating drug resistance to avoid cytotoxic chemotherapy, promote tissue invasion and cell migration by modulating transcription factors, miRNA processing and chromatin remodelling^13–19^. In the case of prostate and breast cancer, LMTK2 and LMTK3 respectively can contribute to cancer development by interacting with the androgen receptor and oestrogen receptor α and modulating their functions^4,20^.

LMTKs are also extensively studied in the context of endosomal vesicle trafficking and axonal transport as they are present in endosomal membranes and bind to myosin VI and kinesin-1/kinesin light chain molecular motors, and are also involved in microtubule remodelling^21–25^. Specifically, LMTK1 is involved in axonal and dendritic outgrowth and maturation by regulating Rab11-positive endosome transport^26–28^. LMTK2 and LMTK3 also bind to and regulate the transport of several proteins which are all involved in synaptic functions, memory and learning such as α-amino-3-hydroxy-5-methyl-4-isoxazolepropionic acid (AMPA) and N-methyl-D-aspartic acid (NMDA) receptor subunits, potassium-chloride cotransporter 2, and the synaptic kinase cyclin-dependent kinase 5 (cdk5)/p35^21,23,29,30^. Apart from cdk5/p35, LMTKs also interact with protein phosphatase 1 to regulate glycogen synthase kinase-3β activity, which is also linked to synaptic functions^15,31–35^. Recently, both LMTK1 and LMTK2 have been linked to Alzheimer’s disease, amyotrophic lateral sclerosis/frontotemporal dementia and global developmental delay/intellectual disability, neurodegenerative and neurological diseases characterised by axonal transport defects and synaptic loss, suggesting that the role of LMTKs in intracellular transport is an important factor for the pathogenesis of these diseases^1–3,21,36–38^. LMTK2 also binds to and phosphorylates cystic fibrosis transmembrane conductance regulator (CFTR) to regulate its availability on the plasma membrane^6^. CFTR mutations cause cystic fibrosis and LMTK2 might be a potential drug target for the treatment of the disease. The presence and abundance of CFTR targeting LMTK2 in the apical membrane of airway epithelial cells is controlled by Rab11-positive endosomes in a transforming growth factor (TGF)-β1-dependent manner^39^. LMTK2 and TGF-β1 signalling is interwoven in complex signalling cascades as not only TGF-β1 affects LMTK2 signalling but TGF-β1 signalling is also influenced by LMTK2 regulatory network^40^ (Fig. 1).

All these findings direct the spotlight on LMTKs as potential disease drivers and therapeutic targets. However, their several alternative and misleading names cause scientific conundrum. Now that these kinases have gained growing attention, it is imperative to use a simple and unambiguous LMTK nomenclature to avoid further confusion. Following the ‘1^st^ LMTKs international conference 2023’, the authors of this manuscript and members of this consortium set forth a novel lemur kinase nomenclature to rectify this problem and help the scientific community.

## A brief history of lemur kinase nomenclature

The first lemur kinase family member was identified as a gene whose expression increases in mouse myeloid precursor cells during apoptosis. Sequence analysis suggested that it is a novel, putative tyrosine kinase since its kinase domain showed strong homology with other tyrosine kinases, and therefore, it was termed as apoptosis-associated tyrosine kinase (AATYK)^41^. Its human homologue was identified by the Kazusa cDNA sequencing project, an effort to identify novel human genes, and got the identification number *KIAA0641*^42^. The human genes of the other two human family members were identified later under the identification numbers *KIAA1079* and *KIAA1883*^43,44^. Parallel with these discoveries, studies aiming to catalogue human protein kinases classified AATYK and the two newly identified genes as a new family of kinases. One of these studies named the family as AATYK and its members AATYK1 (the originally described AATYK), AATYK2 and AATYK3^45^ while the other study named the family as Lmr consisting of Lmr1 (the originally described AATYK), Lmr2 and Lmr3^46^. In this latter case, Manning and colleagues^46^ did not resolve what Lmr abbreviates for but others, in a later publication, referred to them as human lemur (Lmr) kinases^47^. The name lemur potentially originates from the visual similarity of the long carboxyl-terminal tail of the protein which makes it akin to the tail of the Madagascan lemurs. To increase the confusion, a human homologue of AATYK and a splice variant of KIAA0641 was identified in a yeast two-hybrid screen searching for p35 binding partners and was named as human AATYK short isoform-p35 binding polypeptide (hAATYKs-p35BP)^48^. This was the point when the earlier identified human *KIAA1079* gene-encoded protein and its mouse homologue were identified by the Brautigan, Miller and Tadashi groups independently from each other^34,49,50^. Not surprisingly, these parallel discoveries resulted in three new names for the same protein, kinase/phosphatase/inhibitor-2 (KPI-2), Cdk5/p35-regulated kinase (Cprk) and brain-enriched kinase (BREK).

A few years later, Tomomura and co-workers identified two AATYK splice variants and renamed the original AATYK to AATYK1A, and the newly identified form AATYK1B and termed the other two lemur kinase family members as AATYK2, and AATYK3^51^. Interestingly, the gene encoding AATYK had started to be referred to as *AATK* from 2008 without explaining the reason; see e.g., refs. ^52,53^. The year 2006 marks the first appearance of the now widely used LMTK acronym. It was mentioned in a paper describing an LMTK2 knockout mouse model, however, the authors did not explain the origin of this new name and after mentioning it as an alias they kept calling the gene and protein as Brek^54^. Since that year on, all the afore-mentioned names appeared in the literature but mainly usage of AATYK and LMTK dominated it, although some interesting hybrid names have also arisen such as AATKA and AATKB instead of AATYK1A and 1B^55^. To our surprise, after screening various gene and protein databases such as NCBI, Ensembl and UniProt we found several further LMTK aliases which have never appeared in the scientific literature according to our knowledge such as e.g., ‘CDK5-binding protein’, ‘protein phosphatase 1, regulatory subunit 77’ or TYKLM3. The origin of these aliases is unknown and they clearly do not make navigation easier in the already confusing sea of names and abbreviations of the lemur kinases (Table 1).

**Table 1:** Standardised names for the lemur kinase family.

Standardised gene and protein names	Other names
Lemur tail kinase-1 LMTK1	apoptosis-associated tyrosine kinase (AATK, AATYK) apoptosis-associated tyrosine kinase-1 (AATYK1) lemur tyrosine kinase-1 (LMTK1) serine/threonine-protein kinase LMTK1 human AATYK short isoform-p35 binding polypeptide (hAATYKs-p35BP) KIAA0641 LMR1 CDK5-binding protein^a^ brain apoptosis-associated tyrosine kinase^a^ p35BP^a^ protein phosphatase 1, regulatory subunit 77 (PPP1R77)^a^
Lemur tail kinase-2 LMTK2	apoptosis-associated tyrosine kinase-2 (AATYK2) lemur tyrosine kinase-2 (LMTK2) brain-enriched kinase (BREK) Cdk5/p35-regulated kinase (Cprk) serine/threonine-protein kinase LMTK2 kinase/phosphatase/inhibitor-2 (KPI-2) serine/threonine-protein kinase KPI-2^a^ KIAA1079 LMR2 protein phosphatase 1, regulatory subunit 100 (PPP1R100)^a^ FLJ46659^a^ KPI2^a^
Lemur tail kinase-3 LMTK3	apoptosis-associated tyrosine kinase-3 (AATYK3) lemur tyrosine kinase-3 (LMTK3) serine/threonine-protein kinase LMTK3 KIAA1883 LMR3 TYKLM3^a^ phosphatase 1, regulatory subunit 101 (PPP1R101)^a^

^a^Marks those aliases which have never been referenced to in the scientific literature according to our knowledge.

## Kinase specificity of LMTKs

As mentioned above, when LMTK1 was originally identified computational analysis of its kinase domain predicted that it is a tyrosine kinase^41^. However, experimental data conducted by several laboratories independently have proved that LMTK1, LMTK2 and LMTK3 solely phosphorylate serine and threonine residues and do not target tyrosine residue^34,47,50,56^. These findings strongly suggest that their original name is a misleading misnomer and that LMTKs are actually serine/threonine-specific kinases. Although we acknowledge the possibility that LMTKs might be dual-specific serine/threonine-tyrosine kinases there is no experimental data supporting this hypothesis to date. Despite of all the experimental data showing that LMTKs are serine/threonine kinases it might seem surprising that publications categorising kinases have still classified LMTKs as receptor tyrosine kinases until very recently with only one exception^57^. The reason behind it is that kinase family classifications into phylogenetic trees are based on sequence similarities of their catalytic domains and not on their function, however, we would like to emphasise again that LMTKs are serine/threonine-specific kinases.

## The revised LMTK nomenclature

As a result of the ‘1st LMTKs international conference 2023’, all senior PIs agreed that a non-confusing single nomenclature must be adopted to facilitate research in the field. We constructed and are suggesting using the following rules and rationale.

1. The highly confusing reference to tyrosine kinase activity must be eliminated. At the same time, we suggest keeping the most widely used acronym LMTK. In this line, we embrace a recent suggestion to name these proteins ‘lemur tail kinases’ instead of ‘lemur tyrosine kinases’^58^. This naming approach has already been followed by some recent publications^8,27^.
2. Name lemur kinase genes and proteins identically in agreement with the HUGO Gene Nomenclature Guidelines and the International Protein Nomenclature Guidelines^59^ and https://www.ncbi.nlm.nih.gov/genome/doc/internatprot_nomenguide/. This avoids confusion that exists in the case of LMTK1 where the gene is named as *AATK* while the protein as LMTK1.
3. If new family members are identified, these must bear the name lemur tail kinase (LMTK) and the consecutive Arabic number. New subfamily members should get a Latin capital letter starting with ‘A’.
4. Functional protein-coding splice variants can get their own names but should be clearly distinguished from proteins encoded by different genes to avoid confusion with gene subfamily members using a hyphen and lowercase Latin letters starting with ‘a’. Therefore, we suggest using LMTK1-a and LMTK1-b instead of LMTK1A and LMTK1B for the LMTK1 splice variants.
5. We recommend that a statement needs to be included within the introduction section of publications referring to the former LMTK gene and protein names. For example, LMTK2 is a membrane-anchored serine/threonine kinase formerly known as kinase/phosphatase/inhibitor-2 (KPI-2), Cdk5/p35-regulated kinase (Cprk), brain-enriched kinase (BREK), apoptosis-associated tyrosine kinase-2 (AATYK2), KIAA1079 and LMR2^34,43,46,49–51^.

*Research groups supporting the use of this system for lemur kinase nomenclature include the following:*

Neil A. Bradbury, Oana Caluseriu, Georgios Giamas, Shin-ichi Hisanaga, Heinz-Josef Lenz, Christopher C.J. Miller, Stephen J. Moss, Gábor M. Mórotz, Agnieszka Swiatecka-Urban.
